# Binding of Hyaluronan to the Native Lymphatic Vessel Endothelial Receptor LYVE-1 Is Critically Dependent on Receptor Clustering and Hyaluronan Organization*

**DOI:** 10.1074/jbc.M115.708305

**Published:** 2016-01-28

**Authors:** William Lawrance, Suneale Banerji, Anthony J. Day, Shaumick Bhattacharjee, David G. Jackson

**Affiliations:** From the ‡ MRC Human Immunology Unit, Weatherall Institute of Molecular Medicine, John Radcliffe Hospital, University of Oxford, Oxford OX3 9DS, United Kingdom and; the § Wellcome Trust Centre for Cell Matrix Research, Faculty of Life Sciences, University of Manchester, Manchester M13 9PT, United Kingdom

**Keywords:** cell adhesion, complex, endothelial cell, extracellular matrix, hyaluronan, macrophage, LYVE-1, TSG-6, lymphatic endothelium, transmigration

## Abstract

The lymphatic endothelial receptor LYVE-1 has been implicated in both uptake of hyaluronan (HA) from tissue matrix and in facilitating transit of leukocytes and tumor cells through lymphatic vessels based largely on *in vitro* studies with recombinant receptor in transfected fibroblasts. Curiously, however, LYVE-1 in lymphatic endothelium displays little if any binding to HA *in vitro*, and this has led to the conclusion that the native receptor is functionally silenced, a feature that is difficult to reconcile with its proposed *in vivo* functions. Nonetheless, as we reported recently, LYVE-1 can function as a receptor for HA-encapsulated Group A streptococci and mediate lymphatic dissemination in mice. Here we resolve these paradoxical findings and show that the capacity of LYVE-1 to bind HA is strictly dependent on avidity, demanding appropriate receptor self-association and/or HA multimerization. In particular, we demonstrate the prerequisite of a critical LYVE-1 threshold density and show that HA binding may be elicited in lymphatic endothelium by surface clustering with divalent LYVE-1 mAbs. In addition, we show that cross-linking of biotinylated HA in streptavidin multimers or supramolecular complexes with the inflammation-induced protein TSG-6 enables binding even in the absence of LYVE-1 cross-linking. Finally, we show that endogenous HA on the surface of macrophages can engage LYVE-1, facilitating their adhesion and transit across lymphatic endothelium. These results reveal LYVE-1 as a low affinity receptor tuned to discriminate between different HA configurations through avidity and establish a new mechanistic basis for the functions ascribed to LYVE-1 in matrix HA binding and leukocyte trafficking *in vivo*.

## Introduction

The lymphatic network is the primary anatomical location of the adaptive immune system, within which antigen presenting cells and other leukocytes migrate from tissues to draining lymph nodes for immune surveillance and for the generation and regulation of cellular immune responses (1–5). In addition, the lymphatics provide fluid drainage and allow for removal of immune complexes and interstitial matrix components that undergo degradation during both normal homeostasis and tissue injury. In particular, the large glycosaminoglycan hyaluronan (HA^2^; GlcNAcβ1–4GlcUAβ1–3)*_n_*) undergoes rapid turnover during inflammation (6–8), and a proportion of its breakdown products are conveyed via lymphatic vessels for terminal degradation in draining lymph nodes (9–11). The major HA receptor in the endothelia of afferent lymphatic vessels and lymph node sinuses is the integral membrane protein LYVE-1 (lymphatic vessel endothelial receptor-1), a member of the Link superfamily closely related to the leukocyte HA receptor CD44 (12–14). Expressed on both the luminal and basolateral surfaces of lymphatic endothelium, LYVE-1 has been proposed to mediate uptake of intermediate and high molecular weight (HMW) HA (13, 15) and to function as a receptor for HA-induced lymphangiogenesis (16, 17). In addition, LYVE-1 has been implicated in controlling the entry and/or migration of leukocytes in lymphatic vessels (11, 18–20), based on parallels with the role played by CD44 in similar processes in the blood vasculature (21–24). Curiously, however, although recombinant LYVE-1 in transfected 293T fibroblasts and certain other non-lymphoid backgrounds has been shown to bind HMW HA efficiently, the native receptor in lymphatic endothelium displays little if any HA binding when assayed either *in vitro* or *ex vivo* (12, 13). Although evidence suggests an interaction between HA or HA degradation products and LYVE-1 in lymphatic endothelial cells can transduce downstream signaling and cell proliferation, the interaction is of too low an affinity for detection by conventional imaging techniques (16, 17, 25). The molecular basis for this disparity in HA binding affinity between LYVE-1 in lymphatic endothelium and non-lymphoid cell transfectants is not fully clear. Nevertheless, one important mechanism appears to be a cell lineage-specific sialylation of LYVE-1 in LECs that interferes with HA binding through charge repulsion (11, 27), a feature that has been well documented for CD44 in mononuclear cells and lymphocytes (28–32). Whereas the capacity of CD44 to bind HA can be “unmasked” in such cells through activation of an endogenous membrane-bound sialidase activity by inflammatory cytokines or antigen receptor engagement (33–36), no physiological conditions have yet been identified that unmask HA binding in LYVE-1.

Remarkably, we found recently that HA within the capsule of Group A streptococci, the pathogens responsible for tonsillitis and necrotizing fasciitis, can bind efficiently to LYVE-1 in lymphatic endothelium and that the receptor mediates not only adhesion of these microbes to lymphatic vessels but also lymphatic dissemination in a mouse model of streptococcal soft tissue infection (37). Here we have explored the critical parameters required for uncovering the latent HA binding capacity of native LYVE-1 and present important new data that provide a clearer understanding of its molecular basis. In particular, we show that as a consequence of its weak HA binding affinity (14), LYVE-1 is highly dependent on receptor surface density to support stable interactions with the free glycosaminoglycan through avidity, insofar as binding to HMW HA can be induced in native lymphatic endothelium either through lentivirus-mediated LYVE-1 overexpression or mAb-induced local clustering. Moreover, in partial analogy with CD44 (39, 40), we show that binding to native LYVE-1 can also be induced by prior organization of HMW HA as bHA·streptavidin multimers or as cross-linked complexes with the inflammation associated matrix-reorganizing protein TSG-6 (41, 42), most likely through the capacity of such complexes to recruit LYVE-1 in surface clusters. Finally, we show that HA assembled on the surface of macrophages, like that in the surface capsule of Group A streptococci, can interact with endogenous LYVE-1 in lymphatic endothelium *in vitro* and support transendothelial migration. These properties identify LYVE-1 as a highly regulated HA receptor that is tuned to bind its ligand selectively, when organized in an appropriate HA configuration, and provide new insight into the molecular mechanisms regulating LYVE-1 ligand interactions in inflammation and immunity.

## Experimental Procedures

### 

#### 

##### Primary Lymphatic Endothelial Cells and Immortalized Cell Lines

Primary human dermal lymphatic endothelial cells (HDLEC) were isolated from the skin of healthy adults undergoing elective plastic surgery at the John Radcliffe Hospital (Oxford, UK) as described previously (43) with full United Kingdom ethical approval. Briefly, skin was digested overnight at 4 °C with Dispase® (2 mg/ml; Calbiochem) in PBS, and dermal cells were recovered by scraping, followed by passage through a 70-μm cell strainer, prior to initial adherent culture in 0.1% gelatin-coated flasks in complete medium (EGM-2 MV; Lonza). Cells were then lifted with Accutase® (PAA Laboratories), and HDLEC were immunoselected using mouse anti-human LYVE-1 mAb 8C and anti-mouse IgG MACS® magnetic bead preparations (Miltenyi Biotec) followed by culture in 0.1% gelatin (Sigma)-coated flasks in EGM-2 MV. Cultures were used at early passage (≤5) and consistently displayed authentic expression of lymphatic endothelial cell markers Prox-1, LYVE-1, podoplanin, and CD31 and absence of blood endothelial cell markers PAL-E and CD44, as assessed by immunofluorescence microscopy and flow cytometry on a Cyan ADP Analyzer (Dako) using standard parameters.

HEK293T human fibroblasts and the human Jurkat T cell line used for LYVE-1 transfections were obtained from the Cancer Research UK cell bank (Clare Hall, London, UK) and as a kind gift from Prof. S. J. Davis (Weatherall Institute of Molecular Medicine, University of Oxford), respectively.

##### Recombinant TSG-6 Proteins

Full-length recombinant human TSG-6 (rhTSG-6, residues 18–277 of the preprotein) and a truncated form of the protein lacking the C-terminal CUB_C domain (Link_TSG6, residues 36–133) were expressed and purified as described previously (44–46).

##### Antibodies

Polyclonal hLYVE-1 antibody and the monoclonal non-HA-blocking antibodies 6A, 7C, and 8C that bind overlapping epitopes in hLYVE-1 (data not shown) were generated by immunizing rabbits and mice, respectively, with soluble recombinant hLYVE-1 ectodomain (Met^1^–Ser^232^) fused to IgFc as described previously (15). The commercial non-HA-blocking hLYVE-1 mAb 20892 and the commercial HA-blocking mAbs 20891 and 20893 (abbreviated to 892, 891, and 893, respectively) and goat polyclonal hLYVE-1 antibody are each generated against soluble recombinant hLYVE-1 ectodomain (Ser^24^–Thr^238^) and were obtained from R&D Systems. The precise epitopes for these LYVE-1 mAbs are as yet undefined. Other antibodies, including JC70A (hCD31), were from Dako, NZ-1 (hPodoplanin) was from AngioBio, BRIC235 (hCD44) was from IBGRL, and goat polyclonal podoplanin was from R&D Systems.

##### Preparation of Monovalent Fab Fragments

Fab fragments of hLYVE-1 mAbs 8C and 6A were generated by Ficin digest according to the manufacturer's instructions (mouse IgG_1_ Fab preparation kit, Pierce). Samples of intact antibody and Fab fragments were further purified by size exclusion chromatography using a Superdex S200, 10/300 column (GE Healthcare). Fractions spanning the appropriate peaks were pooled and analyzed for purity by non-reducing lithium dodecyl sulfate-PAGE.

##### LYVE-1 Overexpression by Lentiviral Gene Transduction

Full-length hLYVE-1, mouse LYVE-1, or LYVE-1 GFP cDNAs (12, 13) were cloned into the lentiviral vectors, either pHR SIN for high level expression under the CMV promoter and HIV long terminal repeat (LTR) or pHRI SIN for lower level expression under the glucocorticoid response element promoter (47). After co-transfection of HEK 293T cells with a mixture (20 μl aqueous, 100 μl of DMEM, and 4.5 μl of Genejuice® transfection reagent (Merck Millipore)) comprising the appropriate pHR/pHRI construct (0.5 μg) and the packaging plasmids p8.91 ExQV (0.5 μg) (which encodes the viral Gag, Pol, Rev, and Tat proteins) and pMD-G (0.5 μg) (which encodes the VSV-G envelope protein), secreted virus was harvested by centrifugation (500 × *g*, 5 min) and passed through a 0.45-μm filter. The resulting viral supernatant was then used to transduce either HDLEC monolayers (50% confluence) plated in 6-well tissue culture dishes or Jurkat T cells (1 × 10^6^ cells in 2–4 ml), and cells expanded in culture prior to selection for high LYVE-1 expressors using a MoFlo^TM^ high-speed fluorescence-activated cell sorter (Beckman Coulter) run with standard parameters.

##### Preparation of Biotinylated HA

For biotinylation, high molecular weight HA from rooster comb (5 mg/ml; Sigma) in 0.1 m MES, pH 5.5, was combined with EDAC in 0.1 m MES, pH 5.5 (6.25–25 mg/ml) to a final concentration in the range of 81.3–325 μg/ml. Biotin-LC-hydrazide (50 mm; Pierce) in DMSO was then added to a final concentration of 1 mm, and the mixture was stirred overnight (25 °C) before dialysis against 0.05% (w/v) aqueous NaN_3_. Samples were validated for binding to hLYVE-1-transfected Jurkat cells and stored at 5 °C prior to use.

##### Measurement of Constitutive and mAb-induced HA Binding

**FIGURE 1.**
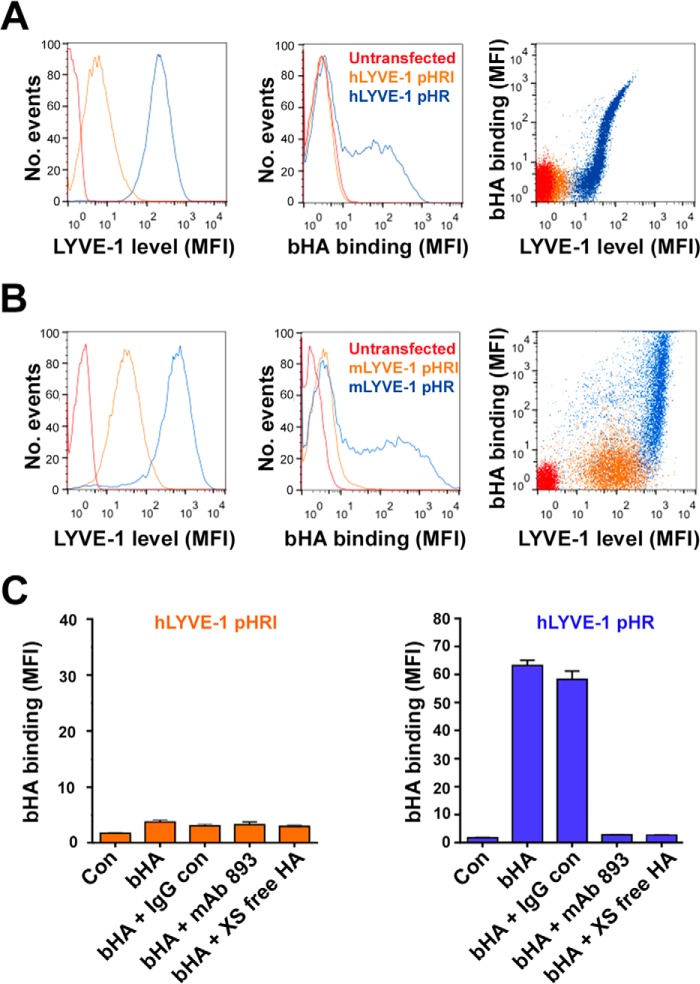
**LYVE-1 surface expression level is critical for HA binding in transfected Jurkat cells.** The bHA binding capacity of Jurkat cells expressing either low/intermediate or high levels of human or murine LYVE-1 following lentiviral transduction with full-length cDNAs in the vectors pHRI and pHR, respectively, was assessed using flow cytometry. *A*, representative individual FACS histograms for hLYVE-1 surface expression as mean fluorescence intensity (*MFI*; LYVE-1/Alexa 488 conjugate) and bHA binding levels (streptavidin Alexa 647) with number of events normalized as described under “Experimental Procedures” and two-color FACS plots depicting bHA binding as a function of receptor expression for control untransfected and hLYVE-1 pHRI- and hLYVE-1 pHR-transduced cells, as indicated. *B*, a similar experiment for mouse LYVE-1-transduced Jurkat cells. *C*, a quantitative analysis of bHA binding for hLYVE-1 pHRI- and pHR-transduced cells and the effects of competition with the hLYVE-1 HA-blocking mAb 893, isotype-matched control IgG, or a 20-fold excess of unlabeled HA, as indicated. Values are the mean fluorescence intensity ± S.D. (*n* = 3) from an individual representative experiment that was repeated twice.

**FIGURE 2.**
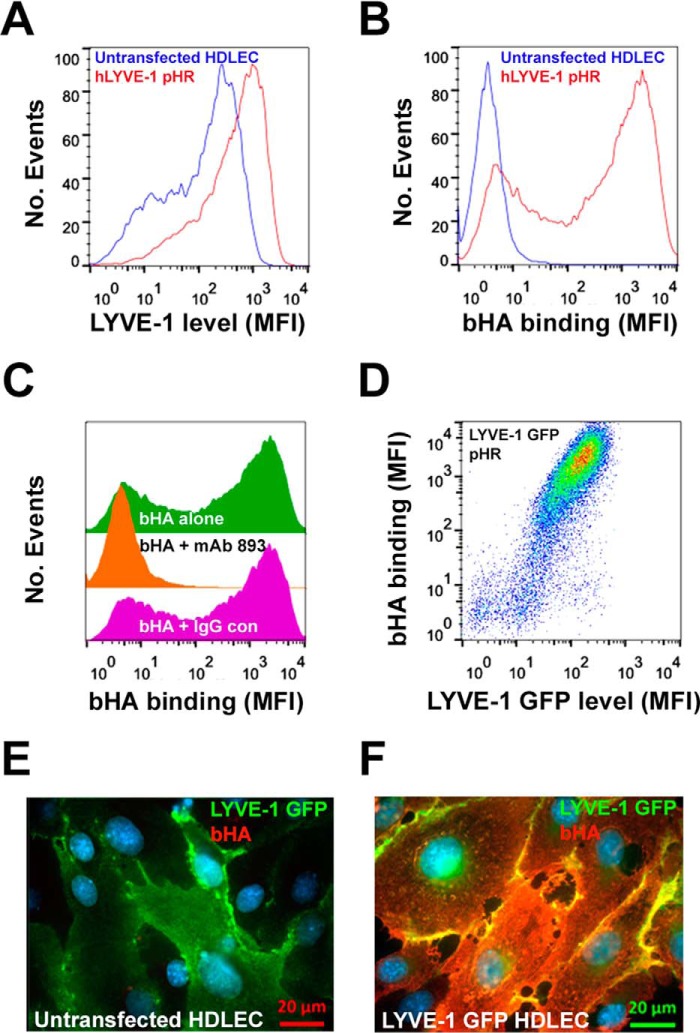
**LYVE-1 surface expression level dictates HA binding in primary lymphatic endothelial cells.** The bHA binding capacity of primary HDLEC expressing either normal endogenous levels or high levels of hLYVE-1 following lentiviral transduction with full-length cDNA in the vector pHR was assessed by flow cytometry. *A*, a representative FACS histogram of hLYVE-1 surface expression as mean fluorescence intensity in control (untransfected) and LYVE-1 pHR-transfected cells. *B* and *C*, levels of bHA binding as well as the effects of competition with the hLYVE-1 HA-blocking mAb 893 or an isotype-matched control IgG, respectively. The numbers of events analyzed were normalized as described under “Experimental Procedures.” *D*, a two-color FACS plot, depicting bHA binding as a function of hLYVE-1 surface expression in HDLEC transduced with an hLYVE-1 GFP fusion construct in the pHR vector (see “Experimental Procedures”). *E* and *F*, fluorescence microscopy images of control untransfected and hLYVE-1 GFP pHR-transduced HDLEC monolayers that had been incubated with bHA prior to fixation and detection with streptavidin Alexa 647 (*red*) and LYVE-1/Alexa 488-conjugated Ab (*green*).

**FIGURE 3.**
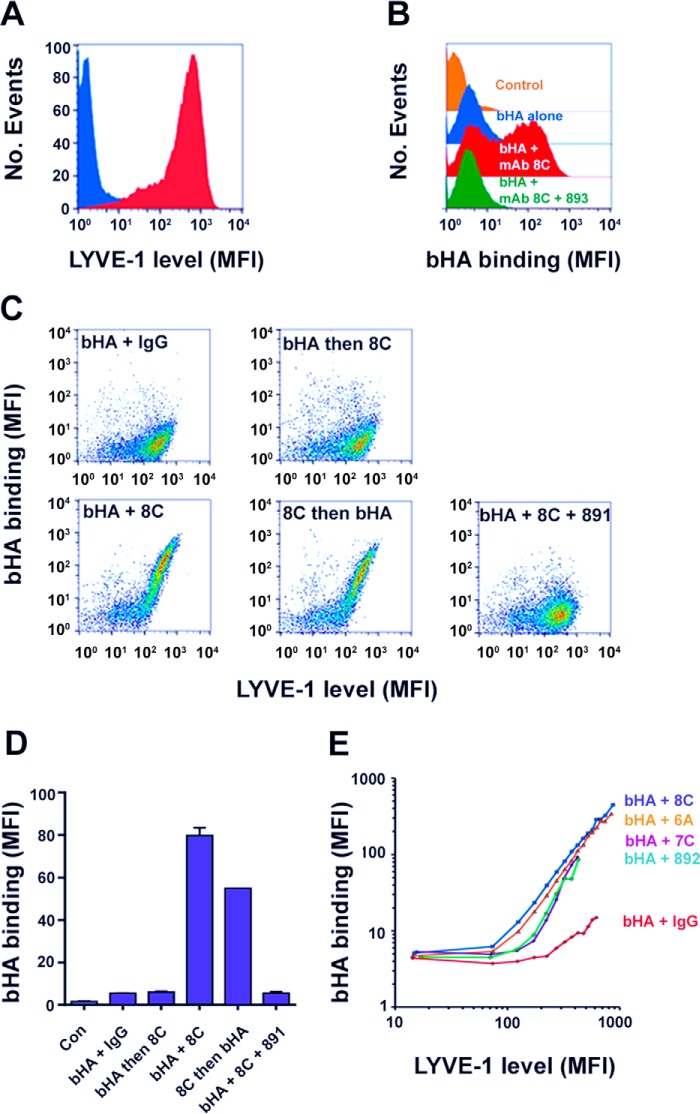
**Antibody-mediated cross-linking of LYVE-1 triggers HA binding in native untransfected lymphatic endothelial cells.** The effects of LYVE-1 cross-linking on bHA binding were assessed by incubating suspensions of live unfixed and untransfected HDLEC with bHA together with bivalent hLYVE-1 mAbs under various different conditions as indicated in the figure. *A* and *B* show representative FACS histograms for HDLEC after initial staining for LYVE-1 (*blue*, control IgG; *red*, LYVE-1/Alexa 488 conjugate; *A*) or following incubation in the presence of bHA either alone or together with the hLYVE-1 mAb 8C or isotype-matched control IgG or a combination of mAb 8C and the hLYVE-1 HA-blocking mAb 893, prior to fixation and detection with streptavidin Alexa 647- and Alexa 488-conjugated Abs as appropriate. *C* and *D*, the effects on HA binding of incubating HDLEC with either bHA and IgG (control); bHA alone, followed by washing and reincubation with mAb 8C; bHA and mAb 8C together; mAb 8C alone, followed by washing and reincubation with bHA; or bHA + mAb 8C + HA-blocking mAb 891 added together. Data are shown as two-color FACS analyses for bHA binding as a function of LYVE-1 surface level (*C*) and as a bar chart in which the binding data are analyzed quantitatively (*D*; data are mean ± S.D. (*error bars*), *n* = 3). The graph in *E* shows the levels of bHA binding in relation to LYVE-1 surface expression in HDLEC co-incubated with bHA and the individual hLYVE-1 mAbs indicated or control IgG prior to analysis by flow cytometry. Values are mean fluorescence intensity (*MFI*) of bHA binding (streptavidin Alexa 647) within set gated intervals of LYVE-1 surface expression (mean fluorescence intensity; Alexa 488-conjugated Ab). Data shown are in each case from single representative experiments that were repeated at least twice.

For quantitation of HA binding, cells (HDLEC- or hLYVE-1 pHR-transfected Jurkat) were resuspended in FACS buffer (5% FCS, 5 mm EDTA, 0.05% NaN_3_ in PBS) and incubated with biotinylated HA (5 μg/ml) either alone or in combination with LYVE-1 mAbs (5 μg/ml), isotype-matched control Ig (5 μg/ml), or excess unlabeled HA (200 μg/ml) for 30 min at room temperature. Cells were then washed (three times) and reincubated in buffer containing appropriate Alexa 488-conjugated secondary antibody (10 μg/ml) and streptavidin Alexa 647 (5 μg/ml) for 30 min at 4 °C, followed by fixation in 2% (w/v) formaldehyde, 0.02% NaN_3_ in PBS and analysis by flow cytometry (Cyan ADP Analyzer, Dako). The numbers of events analyzed in FACS plots (see Figs. 1–3) were normalized using proprietary Summit version 4.3 software. In some experiments, the concentration of LYVE-1 mAb was varied (0–800 nm) to determine the relationship between the extent of receptor cross-linking and bHA binding. In others, the cells were incubated with bHA either before or after cross-linking with various LYVE-1 mAbs, with three washes between incubations, to establish the importance of sequence order. All other parameters were maintained as described.

##### Imaging of mAb-induced LYVE-1 Surface Clustering

Primary untransfected HDLEC cultured on gelatin-coated 8-chamber slides were first blocked with 5% (v/v) FCS, 0.05% (w/v) NaN_3_ in PBS prior to incubation with either bHA alone (5 μg/ml), hLYVE-1 mAbs/Fabs (7.5 μg/ml), or both components together for 30 min at room temperature. Cells were then fixed with freshly depolymerized paraformaldehyde and reblocked prior to detection of LYVE-1 with goat anti-hLYVE-1 polyclonal Ab/Alexa conjugate and of bound bHA with streptavidin Alexa 488 (5 μg/ml), respectively. After an additional final fixation, cells were stained with DAPI and mounted in Vectashield® for imaging on a Zeiss Axiovert S100 inverted epifluorescence microscope fitted with a ×100 oil immersion objective and a Hammamatsu Orca digital camera.

##### Measurement of Streptavidin·bHA Multimer Binding

To prepare multimers, bHA (5 μg/ml) was initially added to streptavidin Alexa 647 (SA647) over a range of concentrations from 0.002 to 20 μg/ml for 30 min at room temperature to determine the optimal bHA/SA647 ratio for LYVE-1 binding. Subsequently, complexes were prepared using a ratio of 2:1 (w/w) by mixing bHA (10 μg/ml) and SA647 (5 μg/ml). For binding studies, hLYVE-1 pHR-transfected Jurkat cells or HDLEC were resuspended in FACS buffer (PBS, pH 7.5, supplemented with 5% (v/v) FCS, 5 mm EDTA, and 0.05% w/v NaN_3_) and incubated with bHA·streptavidin Alexa 647 multimers or control uncomplexed bHA (10 μg/ml) either alone or in combination with LYVE-1 HA-blocking mAb 891 (20 μg/ml) or an excess of unlabeled HA (200 μg/ml) for 40 min at 4 °C. After washing (three times), cells were fixed, and the amount of bound HA·SA647 multimer was quantitated by flow cytometry using a Cyan ADP Analyzer (Dako).

##### Measurement of TSG-6·HA Complex Binding

For preparation of complexes, biotinylated HA (200 μg/ml) was mixed with either full-length rhTSG-6 or the isolated Link module (Link_TSG6) at ratios of 5:1–10:1 protein/HA (w/w) in 40 mm Na-HEPES buffer, pH 6.0, and incubated for 30 min at 25 °C as described previously (39, 48). Resulting TSG-6·HA complexes were then diluted into FACS buffer at a concentration of 2 μg/ml HA and either used immediately or stored at −20 °C.

For experiments with hLYVE-1 pHR-transfected Jurkat cells, these were incubated at 2 × 10^6^ cells/ml in buffer for 10 min at 4 °C. Cells were then incubated with bHA alone or bHA·TSG-6 complexes at a range of HA concentrations, for 30 min at room temperature. Cells were washed three times with FACS buffer and incubated with streptavidin Alexa 647 (5 μg/ml) in FACS buffer for 30 min at room temperature prior to washing and resuspension in FACS fixative. The fluorescence intensity was then measured for 20,000 cells/sample using flow cytometry (Cyan ADP Analyzer, Dako).

For experiments with primary HDLEC, native untransfected cells or LYVE-1 pHR/pHRI-transfected cells, as appropriate, were lifted with Accutase®, washed, and then incubated in buffer for 10 min at 4 °C. Cells were divided into samples of ∼250,000 cells and incubated with bHA alone (5 μg/ml), bHA and TSG-6 (5 μg/ml bHA and 50 μg/ml Link_TSG6 or 25 μg/ml rhTSG-6, mixed immediately prior to incubation), or bHA·TSG-6 complexes either alone or in combination with LYVE-1 mAbs (10 μg/ml), isotype-matched controls (10 μg/ml), or an excess of unlabeled HA (200 μg/ml) for 40 min at 4 °C. Cells were washed three times with buffer and incubated with streptavidin Alexa 647 (5 μg/ml) in buffer for 30 min at 4 °C. Cells were subsequently washed three times with buffer and resuspended in fixative. The fluorescence intensity was then measured for 20,000 cells/sample using flow cytometry (Cyan ADP Analyzer, Dako).

##### Microscopic Imaging of TSG-6·HA Complex Binding to Primary HDLEC

Primary HDLEC were cultured on gelatin-coated 8-chamber slides. Complexes of bHA and Link_TSG6 or rhTSG-6 were formed as described above. Cells were blocked with 5% (v/v) fetal calf serum (FACS buffer) for 5 min at room temperature and then incubated with bHA, bHA-Link_TSG6, or bHA-rhTSG-6 (5 μg/ml) in buffer for 30 min at room temperature. Cells were washed five times with FACS buffer and fixed for 20 min at room temperature. Cells were washed three times with buffer and incubated with streptavidin Alexa 647 (5 μg/ml) and Alexa 488-conjugated 8C mAb (10 μg/ml) for 30 min at room temperature. Cells were washed five times with buffer, fixed once more, and mounted with Vectashield® (Vector Laboratories) mounting medium with DAPI, to stain nuclei, and a coverslip. Images were captured using an inverted epifluorescence microscope (Zeiss Axiovert S100) fitted with a ×40 objective and a Hammamatsu Orca digital camera.

##### Isolation and Culture of Human Peripheral Blood Macrophages

Macrophages were prepared by *in vitro* differentiation of peripheral blood CD14^+^ monocytes, isolated from commercial human leukocyte-enriched blood preparations (leukocyte cones, United Kingdom National Health Service Blood and Transplant Services). Briefly, leukocyte cones were diluted to a volume of 50 ml in PBS and layered overLymphoprep^TM^ density gradients (Stemcell Technologies) followed by centrifugation at 800 × *g* for 20 min to yield the mononuclear cell fraction. Monocytes were then immunoselected on human CD14 MACS® microbeads by magnetic retrieval according to the manufacturer's instructions (Miltenyi Biotec). The resulting CD14^+^ monocytes were cultured as adherent cells in RPMI 1640 medium (Sigma) supplemented with 10% FCS (Sigma), 100 IU/ml penicillin and streptomycin (Sigma), 2 mm l-glutamine (Sigma), and 50 ng/ml GM-CSF (R&D Systems) for 6 days at 37 °C to generate monocyte-derived macrophages (MDM). To polarize macrophages toward an activated M1 phenotype, differentiated cells were stimulated with 1 μg/ml LPS from *E. coli* O55:B55 (Sigma) and 20 ng/ml interferon-γ for 24 h. Expression of macrophage markers (CD11b, CD68, and CD14) was confirmed by flow cytometry.

##### Measurement of in Vitro Macrophage Adhesion and Transendothelial Migration

For adhesion assays, LPS and interferon-γ-activated MDM (see above) were labeled with CellTracker^TM^ Green dye (Life Technologies, Inc.) by incubation (37 °C, 5% CO_2_) for 40 min in EGM II medium supplemented with 10% (v/v) rabbit serum. Labeled cells (5 × 10^5^) were then either left untreated or digested (30 min at room temperature) with *S. hyaluroticus* hyaluronidase (10 units/ml; Sigma), washed, and layered over confluent resting or TNFα (10 ng/ml, 12 h) activated HDLEC monolayers in 24-well tissue culture plates for 2–6 h in the same medium with or without LYVE-1-cross-linking or function-blocking mAbs or isotype-matched control IgG. Wells were again washed (four times) in EGM II medium, and adherent cells were detected and enumerated using a Biotek Synergy HT fluorescence plate reader.

For transmigration assays, CellTracker^TM^ Green-labeled MDM (5 × 10^5^ cells) were incubated for periods of up to 6 h (room temperature) over resting or TNFα-activated HDLEC monolayers (5 days postconfluence) plated on the undersurface of 3-μm pore Fluoroblock® filters mounted in transwell inserts (BD Falcon) essentially as described previously (43, 49). Briefly, inserts with HDLEC were either left untreated or in some cases treated (30 min) with *S. hyaluroticus* hyaluronidase (10 units/ml; Sigma) to digest surface-associated HA, followed by washing. For experiments that investigated the effects of LYVE-1 cross-linking or functional blockade, LYVE-1 mAb 8C, 6A, or 893 or isotype-matched control IgG was then added to HDLEC-coated inserts at a final concentration of 20 μg/ml, and numbers of transmigrated cells in the bottom chambers were monitored at 10-min intervals using a BioTek Synergy HT fluorescence plate reader.

## Results

### 

#### 

##### Binding of HA to LYVE-1 Is Critically Dependent on Receptor Expression Level and Requires a Minimum Threshold Surface Density

The failure of endogenous LYVE-1 to support stable binding of HMW HA on the surface of primary lymphatic endothelial cells is reminiscent of the closely related receptor CD44, which displays similar functional latency in lymphocytes and monocytes and certain hemopoietic cell lines (21, 33, 35, 50). Both receptors also share weak affinity for HA (*K_d_* 10^−4^ to 10^−5^ m) (27, 32, 51), and in the case of CD44, it is evident that stable binding requires simultaneous engagement of individual HA polymers by multiple receptor molecules (*e.g.* see Ref. 52). Moreover, previous studies using antibody-induced clustering of CD44 or manipulation of CD44 spatial organization in supported lipid bilayers have shown that surface density can be critical for HA binding to this receptor (52–54).

To probe the influence of receptor surface density on LYVE-1/HA interactions, we first transfected Jurkat T cells with full-length hLYVE-1 cDNA in the lentiviral plasmids pHR SIN and pHRI SIN that yield high and intermediate levels of LYVE-1 expression, respectively, and determined the consequences for binding to HMW bHA by flow cytometry. This particular cell line lacks endogenous expression of both LYVE-1 and CD44 but replicates the low HA binding state of native HDLEC on transfection with LYVE-1, as reported previously (27). As shown in Fig. 1*A*, transduction with the hLYVE-1 pHRI SIN construct yielded low to intermediate levels of LYVE-1 surface expression, whereas transduction with the pHR SIN construct yielded LYVE-1 levels that were almost an order of magnitude higher. Significantly, however, bHA binding was only manifest among cells transduced with the LYVE-1 pHR construct. Furthermore, as was evident from the two-color FACS plots in Fig. 1 (*A* and *B*), the process required a minimum threshold level of LYVE-1 expression, above which binding appeared to increase almost exponentially with receptor density. Importantly, the interaction could be fully disrupted either by competition with the LYVE-1 HA-blocking mAb 891 or with an excess of unlabeled HA (Fig. 1*C*). Comparable results were obtained when Jurkat cells were transduced with mouse LYVE-1 pHR (Fig. 1*B*); indeed, in this case, the higher expression levels attained were associated with a proportionately greater increase in bHA binding.

Next, to determine the influence of LYVE-1 density in a native endothelial cell background, we assessed bHA binding in lentivirally transduced primary HDLEC. As expected, normal untransfected HDLEC displayed moderate but characteristically heterogeneous levels of LYVE-1 surface expression and bound little if any HMW bHA (Fig. 2, *A* and *B*). However, following transduction with LYVE-1 pHR SIN, which yielded a 10-fold increase in mean surface expression, the cells displayed extensive HMW bHA binding, and this was fully inhibited by the hLYVE-1 HA-blocking mAb 893 (Fig. 2*C*), validating the specificity of the interaction. Moreover, when HDLEC were lentivirally transduced with a C-terminal GFP-tagged LYVE-1 construct to allow its distinction from endogenous receptor, the requirement of a critical threshold density for HA binding was even more apparent (Fig. 2*D*). Indeed, imaging of LYVE-1 pHR SIN-transduced HDLEC monolayers that had been incubated with bHA showed the glycosaminoglycan bound over the entire cell surface although more abundantly at cell-cell junctions where LYVE-1 expression was highest (Fig. 2, *E* and *F*). These results establish that HA binding in lymphatic endothelium is not only dependent on LYVE-1 surface density but also requires a minimum critical threshold level to stabilize the interaction.

##### Binding of HA to Native LYVE-1 in Lymphatic Endothelium Is Induced by Receptor Clustering

**FIGURE 4.**
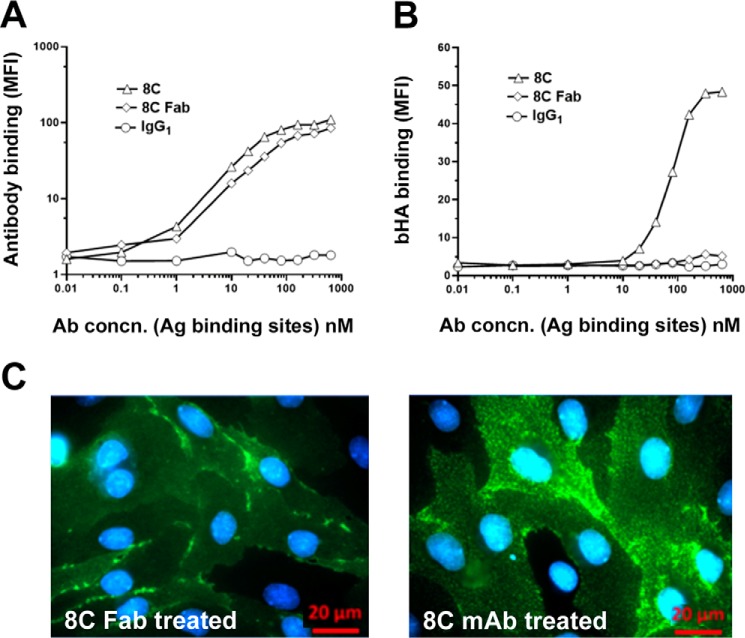
**Critical dependence on LYVE-1 antibody bivalency for mAb-induced HA binding.** The importance of LYVE-1 antibody valency for induction of bHA binding in primary untransfected HDLEC was investigated by comparing the potencies of intact bivalent 8C mAbs and their monovalent Fab fragments (see “Experimental Procedures”). *A*, a comparison between 8C mAbs, 8C Fab fragments, and control isotype-matched IgG for binding to endogenous LYVE-1 in HDLEC over the range 10 pm to 1 μm (estimated as Ag binding sites) as determined by flow cytometry with Alexa 488-conjugated secondary Abs. *B*, potencies of 8C mAbs, 8C Fab fragments, and control IgG for induction of bHA binding determined by co-incubating HDLEC with bHA and each Ab species over the same concentration range as in *A*, prior to fixation and detection with streptavidin Alexa 647 conjugate (data are mean ± S.D., *n* = 3). *C*, representative epifluorescence microscopic images of HDLEC monolayers cultured on gelatin-coated 8-chamber slides, showing the effect on LYVE-1 surface distribution of incubation with 8C mAbs and Fabs prior to staining with Alexa 488-conjugated secondary Ab (*green*). Nuclei are stained with DAPI (*blue*). Data shown are in each case from single representative experiments that were repeated at least twice.

Whereas gross manipulation of LYVE-1 expression by lentiviral transduction affirmed the importance of receptor surface density, we were also interested in assessing the effects on HA binding of more localized LYVE-1 clustering. To induce such clustering, we cross-linked native endogenous LYVE-1 on the surface of primary untransfected HDLEC using individual mAbs from a panel that includes the commercial non-HA-blocking hLYVE-1 antibody 892 and the previously described non-HA-blocking hLYVE-1 mAbs 6A, 7C, and 8C that recognize as yet undefined but overlapping epitopes (data not shown). Strikingly, as shown for the example of mAb 8C, incubation with primary HDLEC either prior to or simultaneously with bHA provoked extensive HA binding, whereas inclusion after HA binding and washing or substitution with isotype-matched control Ig had no such effect (Fig. 3, *C* and *D*). Furthermore, the potentiating effect of mAb-induced LYVE-1 cross-linking was concentration-dependent (see Fig. 4*B*) and inhibited both by competition with the HA-blocking mAb 891 and by displacement with an excess of unlabeled HA, underlining its specificity (Fig. 3, *C* and *D*) (data not shown). Similar induction of bHA binding was also observed with mAbs 6A and 7C, whose epitopes within the consensus Link HABD partially overlap that of mAb 8C and with mAb 892, whose precise epitope has yet to be determined (Fig. 3*E*). Moreover, analysis of the induction as a function of LYVE-1 surface expression using two-color flow cytometry demonstrated that the process was once again dependent on a critical threshold receptor density and that HA binding was boosted by a very considerable extent (up to 60-fold; Fig. 3*E*).

To determine whether this mAb-induced HA binding was a direct consequence of physical receptor cross-linking, we next compared the potentiating effects of mAb 8C as an intact bivalent Ig and as monovalent Fab fragments generated by controlled digestion with Ficin. As shown in Fig. 4*A*, the 8C Fab fragments retained the same concentration-dependent binding profile for hLYVE-1 as the intact mAb when assessed by flow cytometry with native lymphatic endothelium. In contrast, the monovalent 8C Fab fragments had a dramatically reduced capacity to induce HA binding, eliciting levels that were at least 10-fold lower than those of the intact bivalent mAb (Fig. 4*B*). Indeed, similar results were obtained when bivalent and monovalent preparations of another potentiating mAb 6A were compared (not shown). Of note, neither the bivalent 8C mAbs nor monovalent Fabs provoked a significant increase in HA binding to immobilized hLYVE-1 ectodomains in plate binding assays (data not shown). Overall, these features are consistent with a mechanism of Ab-induced LYVE-1 clustering and avidity-dependent HA binding that depends on free lateral mobility of the receptor in the plasma membrane rather than alteration of LYVE-1 conformational change or downstream signaling.

**FIGURE 5.**
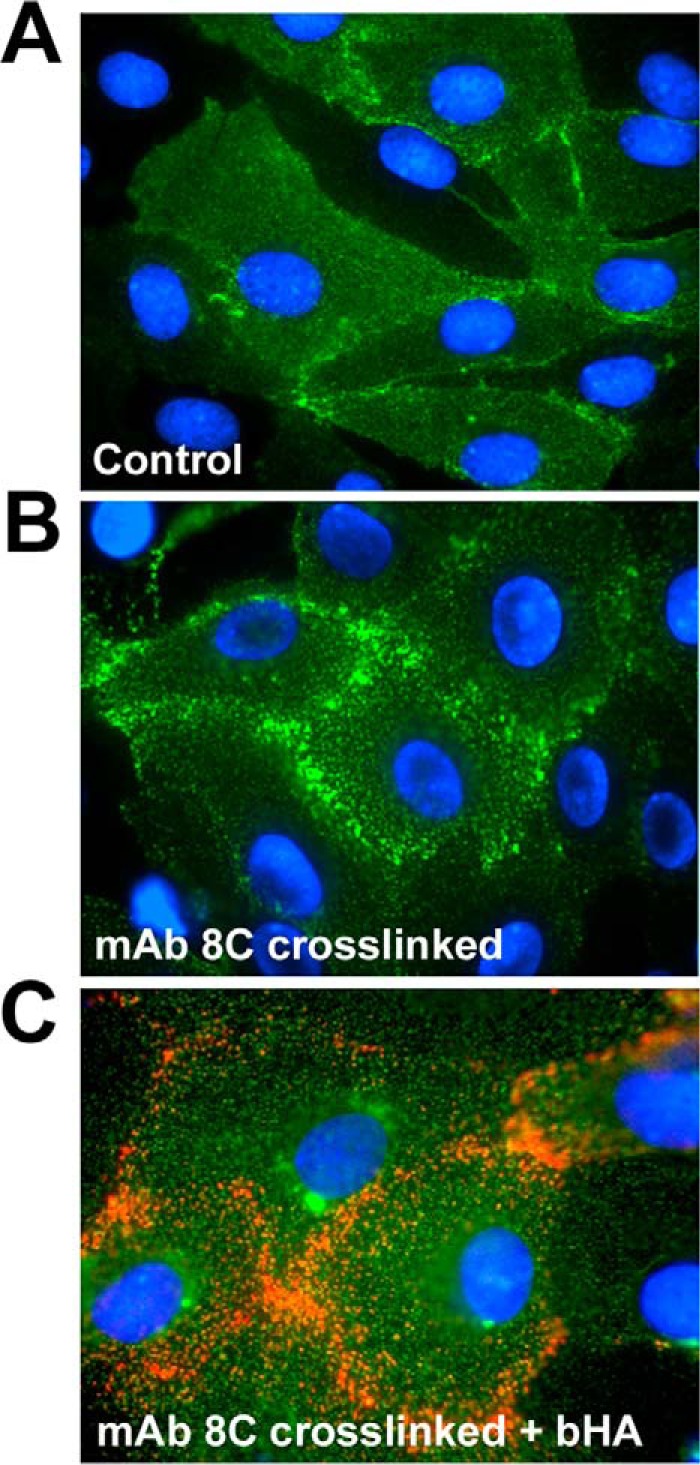
**Antibody-induced cross-linking and bHA binding in HDLEC involves redistribution of LYVE-1 within surface clusters.** The effects on LYVE-1 surface organization of incubating monolayers of primary untransfected HDLEC with bivalent LYVE-1 antibodies were assessed by epifluorescence microscopy. Adherent primary HDLEC were cultured on gelatin-coated 8-chamber slides and either fixed immediately prior to staining with LYVE-1 mAb 8C (*A*, control, no cross-linking) or incubated with mAb 8C and then fixed (*B*) for detection using Alexa 488-conjugated secondary Ab. In addition, HDLEC were co-incubated with mAb 8C and bHA prior to fixation and detection with streptavidin Alexa 647 (*red*) and Alexa 488-conjugated secondary Ab to compare the distribution of LYVE-1 and bound HA (*C*). Nuclei are stained with DAPI (*blue*). Images are from individual representative experiments that were repeated at least twice.

To directly visualize the effects of antibody treatment on LYVE-1 surface distribution, we imaged the receptor on the surface of primary HDLEC monolayers after appropriate incubation, using confocal microscopy. When paraformaldehyde-fixed cells were stained with the hLYVE-1 mAb 8C, the receptor was found to be distributed in a finely punctate pattern over the cell body as well as in more dense aggregates at endothelial cell-cell junctions (Fig. 5*A*). However, when native cells were incubated with mAb 8C prior to fixation with paraformaldehyde and detection with Alexa-labeled secondary Abs, this distribution was altered, and the majority of the receptor was instead organized within discrete clusters close to the cell borders (Fig. 5*B*). Moreover, when native cells were co-incubated with mAb 8C and bHA before fixation and secondary staining, the bound glycosaminoglycan could be seen to associate with these LYVE-1 clusters, particularly near interendothelial junctions, consistent with avidity-induced tethering to the cross-linked receptor (Fig. 5*C*). In contrast, the distribution of LYVE-1 in HDLEC that had been incubated with monovalent 8C Fab fragments was largely unperturbed and resembled the more diffuse ground state of untreated controls (Fig. 4*C*). Together, these findings reveal that HA binding to LYVE-1 in primary HDLEC does not require a gross increase in surface expression but can be induced at normal physiological receptor levels in response to discrete focal clustering.

##### Cross-linking of HA as Multimers or Organization within Supramolecular Complexes with TSG-6 Enables Binding to LYVE-1 in Primary Lymphatic Endothelium

**FIGURE 6.**
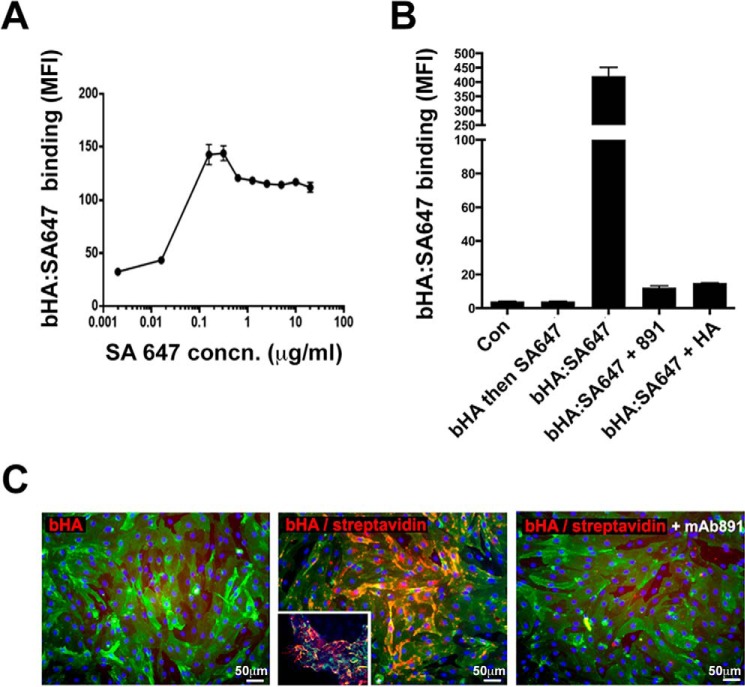
**Multimeric bHA streptavidin complexes can bind LYVE-1 without the requirement for prior clustering.** The effect of cross-linking HA in the form of streptavidin·bHA multimers was investigated using LYVE-1 pHR-transduced Jurkat cells and primary untransfected HDLEC. *A*, a ratiometric titration of complexes generated by premixing increasing concentrations of SA647 and bHA (5 μg/ml), followed by incubation with LYVE-1 pHR-transduced Jurkat cells and quantitation using flow cytometry. *B*, quantitation of bHA binding to primary untransfected HDLEC either when added alone, followed by washing and detection with SA647 (bHA and then SA647), or as a preformed complex (bHA·SA647) with or without the HA-blocking mAb 891 or an excess (20-fold) of unlabeled HA, as indicated. Data are mean ± S.D. (*error bars*), *n* = 4. *C*, fluorescence microscopy images of primary untransfected HDLEC monolayers incubated with bHA alone or as bHA·SA647 complexes with or without the HA-blocking mAb 891 (*green*, LYVE-1; *red*, bHA). The *inset* in the *middle image* depicts an area of the monolayer where the cells spontaneously adopted a tube-like morphology in which the bHA·SA647 complexes bound preferentially. Data shown are in each case from single representative experiments that were repeated at least twice.

Having established the prerequisite of a critical LYVE-1 threshold density for HA binding and shown that LYVE-1 focal clustering can induce the interaction in primary HDLEC, we next investigated whether binding might be equally provoked by organization of HA into higher order structures that might themselves be inducing receptor clustering. To obtain proof of principle, we first generated multimeric complexes by cross-linking bHA with SA647 at various ratios and assessed their binding to LYVE-1 pHR-transduced Jurkat cells by flow cytometry. The results (Fig. 6*A*) showed that multimers generated by mixing bHA (5 μg/ml) with SA647 at concentrations above 0.1 μg/ml (*i.e.* SA647/bHA ratios greater than 0.02:1 (w/w)) provoked extensive binding (up to 5-fold relative to bHA alone). More importantly, when bHA·SA647 (2:1) complexes were incubated with monolayers of primary HDLEC, they displayed up to 30-fold greater binding than if cells were incubated with bHA alone and then washed and reincubated with SA647 (compare *bHA* and *SA647* in Fig. 6*B*). Binding of preformed bHA·SA647 complexes was also fully blocked by inclusion of mAb 891 or by a 20-fold molar excess of free non-biotinylated HA (Fig. 6*B*). Notably, imaging of the monolayers by fluorescence microscopy revealed that bound complexes were distributed over the surface of HDLEC, frequently in discrete clusters that were near endothelial junctions where LYVE-1 is most densely distributed (Fig. 6*C*). Binding was particularly apparent in HDLEC monolayers that occasionally adopted a tubelike morphology resembling lymphatic capillaries *in vivo* (Fig. 6*C*, *inset*). These results demonstrate that native endogenous LYVE-1 can in fact engage HA when the polymer is presented in an appropriately cross-linked state.

**FIGURE 7.**
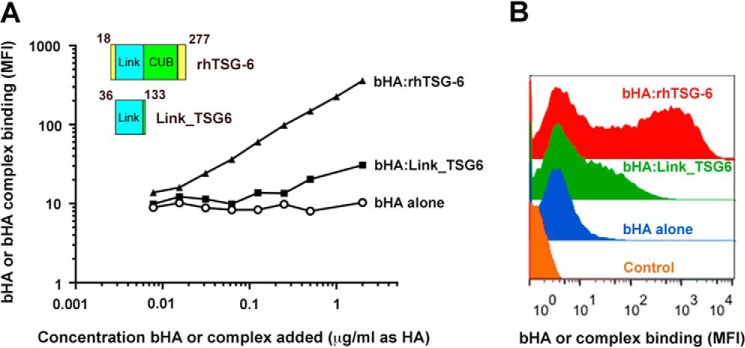
**Precomplexing HA with TSG-6 promotes binding to LYVE-1-transfected Jurkat cells.** The ability of preformed bHA·TSG-6 complexes (5:1 molar ratio) to bind hLYVE-1 in lentivirally transduced Jurkat cells and the importance of multimerization for complex binding were assessed using either rhTSG-6 or the isolated Link domain (Link_TSG6), respectively. *Inset*, comparative structures of the proteins with Link and CUB_C domains in *blue* and *green* and additional sequence in *yellow*; residue numbers are indicated. *A*, a quantitative analysis of bHA·rhTSG-6 and bHA·Link_TSG6 complex binding compared with that of control free uncomplexed bHA over the range of 0.005–2 μg/ml (estimated as HA), as determined by SA647 fluorescence (mean fluorescence intensity (*MFI*)) and flow cytometry. *B*, individual FACS histograms comparing the binding of free bHA and bHA·rhTSG-6 or bHA·Link_TSG6 complexes determined at the maximal concentration used (2 μg/ml). Data shown are in each case from single representative experiments that were repeated at least twice.

Next, to assess the competence of LYVE-1 to bind HA configurations more closely resembling those formed within tissues *in vivo*, we explored the properties of multimeric complexes involving the inflammation-associated HA-binding protein TSG-6, which is composed mainly of contiguous Link and CUB_C domains along with a short N-terminal peptide that supports a wide range of protein and glycosaminoglycan interactions (41, 42, 55, 56). The capacity of this protein to form large HA complexes that are most likely secondarily cross-linked via the CUB_C domains is well documented (40, 42, 48). Most notably, the organization of HA within such TSG-6 complexes has been shown to potentiate its binding to functionally latent states of CD44 in murine leukemic cells (39). Accordingly, we prepared supramolecular complexes of high molecular weight bHA incorporating either rhTSG-6 or the isolated Link module (Link_TSG6) (39). In initial experiments, we assessed binding of each complex to hLYVE-1-transfected Jurkat cells expressing levels of the receptor below the critical threshold for binding to free uncomplexed bHA. As shown in Fig. 7 (*A* and *B*), the cross-linked bHA·rhTSG-6 complexes bound efficiently, and binding was dependent on concentration over the full range tested (5 ng/ml to 2 μg/ml), reaching a maximum level some 30-fold greater than uncomplexed bHA alone. In contrast, bHA·Link_TSG6 complexes displayed much weaker binding that was almost 10-fold lower than that of bHA·rhTSG-6 even at maximal concentrations (Fig. 7), indicating that HA cross-linking mediated by sequence outside of the TSG-6 Link module is critical for engagement with LYVE-1.

**FIGURE 8.**
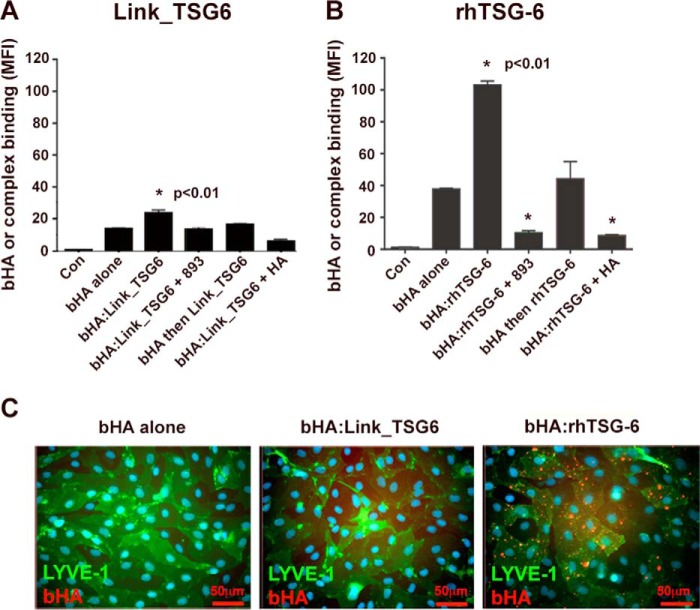
**Cross-linked TSG-6·HA complexes bind efficiently to LYVE-1 in native untransfected HDLEC.** The capacity of TSG-6 to promote HA binding to endogenous LYVE-1 in primary HDLEC was assessed using preformed complexes of bHA with either rhTSG-6 or the isolated Link domain (Link_TSG6) (1:5 molar ratio) by a combination of quantitative flow cytometry and epifluorescent microscopy. *A* and *B*, quantitative analysis of binding for preformed bHA·Link_TSG6 and bHA·rhTSG-6 complexes, respectively, to primary HDLEC compared with that of bHA alone or bHA followed by washing and reincubating with SA647 (bHA and then rhTSG-6/Link_TSG6) with or without the HA-blocking mAb 893 or an excess (20-fold) of unlabeled HA, as indicated. Data are the mean fluorescence intensity (*MFI*) ± S.D. (*error bars*) (*n* = 3). Statistics are a one-way analysis of variance (99% confidence level), using cells incubated with bHA alone as the comparator. *, *p* < 0.01 in each case indicated. *C*, fluorescence microscopic images of primary untransfected HDLEC monolayers incubated with bHA alone, bHA·Link_TSG6, or bHA·rhTSG-6 complexes followed by fixation and staining for LYVE-1 (*green*, Alexa 488 conjugate) and bound bHA (*red*, SA647). Data shown are in each case from single representative experiments that were repeated at least twice.

Remarkably, when incubated with primary HDLEC expressing endogenous native hLYVE-1, bHA·rhTSG-6 yielded a substantial (3-fold) increase in binding relative to free bHA, whereas bHA·Link_TSG-6 binding was again only marginal (Fig. 8, *A* and *B*). This latter result again underlines the importance of the non-Link module region in TSG-6 for potentiating binding of these cross-linked HA complexes to LYVE-1 and marks a distinct contrast with CD44, where its effects were much less apparent (39). Importantly, binding was in both cases fully blocked by mAb 893 and by an excess of free HA, confirming specificity (Fig. 8, *A* and *B*). Intriguingly, imaging of the bound complexes on the surface of HDLEC by confocal microscopy showed that bHA·rhTSG-6 was again largely sequestered within discrete surface clusters, many of which were located at the cell borders, whereas no bHA·Link_TSG6 complexes or (control) free bHA monomers were detected (Fig. 8*C*). These results reveal that HA, when assembled in multimers or organized in appropriately cross-linked supramolecular complexes, can act as ligands for LYVE-1 in lymphatic endothelium, even without prior clustering of the receptor. Moreover, such complexes appear to have the capacity to induce LYVE-1 clustering themselves.

##### HA Present on the Surface of Macrophages Can Bind LYVE-1 in Lymphatic Endothelium to Facilitate Adhesion and Cell Transit

**FIGURE 9.**
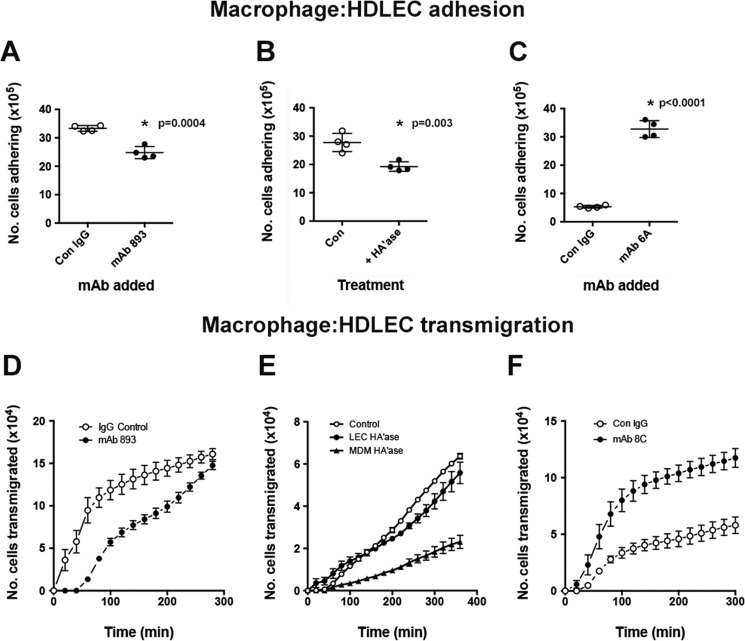
**HA on the surface of macrophages promotes LYVE-1-mediated adhesion to primary HDLEC and transendothelial migration.** The ability of human monocyte-derived macrophages to adhere and migrate across primary HDLEC via LYVE-1 and HA interactions was assessed using static *in vitro* adhesion and transendothelial migration assays (see “Experimental Procedures”). *A*, adhesion of CMFDA-labeled human MDM to TNFα-activated HDLEC monolayers incubated with the HA-blocking mAb 893 or isotype-matched control IgG. *B*, similar adhesion for MDM predigested with hyaluronidase or left untreated (control). *C*, adhesion of MDM to resting HDLEC incubated with the cross-linking mAb 6A or isotype-matched control IgG. Binding was in each case determined using a fluorescence ELISA plate reader; data are the mean ± S.D. (*error bars*) (*n* = 3). *, statistical significance to the indicated *p* values (two-tailed *t* test). *D–F*, representative time courses for migration of CMFDA-labeled monocyte-derived macrophages from the basolateral to luminal face of activated HDLEC monolayers plated on the undersurface of 5-μm pore transwell filters, carried out at 37 °C in the presence of different LYVE-1 mAbs or after hyaluronidase treatment, as indicated. Numbers of transmigrated cells were determined using a fluorescence plate reader. *D*, a time course for transmigration of MDM in the presence of the HA-blocking mAb 893 or control IgG. *E*, a time course for transmigration after hyaluronidase predigestion of either MDM or HDLEC. *F*, a time course for transmigration of MDM in the presence of the cross-linking mAb 8C or control IgG. Values are the mean ± S.D. (*n* = 4). In each case, data shown are from single representative experiments that were repeated at least twice.

Previous reports have indicated that leukocytes, including macrophages, dendritic cells, and T cells, express HA synthases and may even assemble HA matrices on their cell surface (57–61). Hence, we considered the possibility that a leukocyte-bound HA assembly might possess the capacity to bind native LYVE-1 in a manner analogous to the HA·protein complexes described above. Indeed, given our recent finding that the dense HA assembly in the surface capsule of *Streptococcus pyogenes* binds LYVE-1 (37), we considered the possibility that such an assembly might also be configured for LYVE-1-mediated leukocyte adhesion and/or transit of lymphatic endothelium (20). To this end, we prepared MDM by *in vitro* differentiation of human peripheral blood monocytes with GM-CSF, followed by activation with LPS and interferon-γ and fluorescent loading with 5-chloromethylfluorescein diacetate (CMFDA). We then assessed the capacity of these cells to engage in LYVE-1·HA-mediated binding to primary HDLEC using static *in vitro* adhesion assays. As shown in Fig. 9 (*A* and *B*), MDM adhered efficiently to activated HDLEC monolayers during a 2-h incubation. Importantly, however, a small but significant proportion (20–25%) of this binding could be disrupted by the addition of the LYVE-1 HA-blocking mAb 893 (Fig. 9*A*) or by pretreatment of MDM with purified hyaluronidase (Fig. 9*B*). Furthermore, MDM adhesion was enhanced considerably (7-fold) when LYVE-1 was induced to precluster on the surface of resting HDLEC by prior cross-linking with either the 6A or 8C mAbs (Fig. 9*C*) (data not shown), mirroring the enhancement of high molecular weight bHA binding observed earlier with these same antibodies (see Fig. 3). These results indicate that a proportion of HA on the surface of MDM is appropriately organized for adhesion to native LYVE-1 in HDLEC and that the interaction can be further strengthened by prior LYVE-1 surface clustering.

Finally, to investigate the possibility that LYVE-1 engagement with macrophage surface HA might facilitate cell transit across the lymphatic endothelium, we modeled the process using an *in vitro* assay with CMFDA-labeled LPS/interferon-γ-activated MDM and cytokine-activated HDLEC monolayers plated on the undersurface of 3-μm pore Fluoroblok® filters in transwell chambers. The time courses in Fig. 9 (*D–F*) show that MDM transmigrated efficiently over a 5–6-h period, replicating behavior that we observed previously with monocyte-derived dendritic cells (43, 49, 62). More importantly, transit was both reduced by the addition of the LYVE-1 HA-blocking mAb 893 and, in a similar manner to adhesion, was augmented when LYVE-1 was cross-linked in resting HDLEC by preincubation with mAb 8C (Fig. 9, *D* and *F*, respectively). Furthermore, predigestion of macrophages with purified hyaluronidase reduced transmigration by almost 3-fold, whereas similar pretreatment of HDLEC had minimal effect (Fig. 9*E*), confirming the specific involvement of macrophage rather than LEC surface HA. Hence, the rate and extent of MDM transit could be shown to respond in a predictable manner to the inhibition or potentiation of LYVE-1·HA binding by antibody treatment or disruption by hyaluronidase digestion.

In summary, these findings provide a rational molecular explanation for the seemingly contradictory binding properties of LYVE-1 in native lymphatic endothelium, in terms of its apparent incapacity to sequester free HA polymers and its ready ability to engage higher order HA structures, such as the streptococcal surface capsule. In particular, they reveal LYVE-1 as a discriminatory receptor tuned for selective, avidity-dependent interactions with multimeric HA complexes, including those generated during tissue inflammation and those on the surface of leukocytes such as macrophages that can migrate within lymphatic vessels *in vivo*.

## Discussion

Interactions between HA and its cellular receptors are known to be important for uptake of the glycosaminoglycan during normal homeostasis as well as for regulating cell adhesion and migration through the interstitium (63). However, the capacity of cells to tether the glycosaminoglycan is tightly regulated, and not all cells expressing HA receptors can bind HA constitutively (11, 23, 50, 54). More recently, it has also become apparent that HA binding can have diverse functional consequences, depending not only on the size of the bound HA polymers (8, 64) but also the higher order structures they adopt when organized as supramolecular aggregates with specific HA binding partner molecules (39, 40, 65–69).

The endothelial HA receptor LYVE-1 has long been implicated in regulating HA-mediated interactions in the lymphatic system (20). However, despite evidence from detailed biochemical analyses that the recombinant receptor binds HA via a conserved binding site within the N-terminal Link domain *in vitro*, it has not been possible to detect durable binding of either high or low molecular weight HA to the native receptor in primary lymphatic endothelial cells or to lymphatic vessels in freshly resected tissues *ex vivo* (12, 27). In previous studies, we identified endothelium-directed sialylation of LYVE-1 as one important mechanism by which the HA binding affinity of native LYVE-1 is apparently suppressed in LECs and showed that enzymatic cleavage of sialylated sugar chains attached to the membrane-proximal domain could reconstitute binding in LEC lysates (11, 27). However, because no conditions had been identified to trigger LYVE-1 desialylation *in vivo*, this implied somewhat paradoxically that the receptor must be constitutively silenced. Just recently, however, we reported that the dense HA surface capsule of pathogenic Group A streptococcus *S. pyogenes*, the causative agent of tonsillitis and necrotizing fasciitis, can bind LYVE-1 in lymphatic endothelium and that the interaction directs lymphatic dissemination of the microbe, thus indicating that the receptor is in fact active *in vivo* (37).

In this present work, we have presented new data that provide both an explanation for this apparent inconsistency and important insight into the HA binding properties of native LYVE-1 in lymphatic endothelium. Overall, we established that the capacity of native LYVE-1 to bind HA is critically dependent on a combination of receptor distribution and optimal organization of the glycosaminoglycan itself, either in the form of cross-linked supramolecular complexes or as an appropriately configured layer on the surface of a migratory leukocyte. Regarding the first of these, we showed that increasing the surface density of LYVE-1 in primary HDLEC by means of lentiviral transduction allowed binding of high molecular weight HA, thereby revealing the requirement of a critical threshold receptor density for engagement with the sugar polymer. In addition, using mAbs to directly cross-link LYVE-1 in untransfected HDLEC, we showed that HA binding could also be induced by redistribution of the endogenous receptor within discrete surface clusters, a process that occurs rapidly and requires antibody bivalency. In a similar manner to CD44 (52), this dependence on receptor density may be explained by the relatively weak monomer binding affinity of LYVE-1 for its ligand HA (32) and the consequent requirement for multiple receptor molecules to engage individual HA polymers in order to generate sufficient avidity for stable tethering on the endothelial cell surface. Indeed, in a set of independent studies, Dubacheva *et al.* (70, 71) demonstrated that the affinity of a multivalent ligand, such as HA, for its receptor (CD44) renders the binding interaction exquisitely sensitive to changes in receptor density. This phenomenon of “superselectivity” (71) is also likely to explain the sensitive relationship between LYVE-1 surface density and HA binding that we report here. Although not explored in our present studies, we also anticipate that this relationship with LYVE-1 binding avidity will be subject to influence by HA size, a feature that has been documented for interactions with CD44 (52). Moreover, in the context of these new considerations, sialylation of LYVE-1 should no longer be regarded as a modification that “silences” the receptor in LECs as originally envisaged (27) but rather as one that further tunes the avidity of the LYVE-1/HA interaction.

Significantly, we found that mAb-induced LYVE-1 clustering and HA binding could be achieved with a single layer of antibody and did not require secondary cross-bridging. As was evident from microscopic imaging, native LYVE-1 adopts a finely punctate surface distribution in normal resting HDLEC. Hence, it is likely that existing receptor microclusters provide for lattice formation by bivalent mAbs, such as 6A, 7C, and 8A, that bind to single polypeptide epitopes present within the Link domain of each receptor, independent from the HA binding surface. Besides inducing LYVE-1 cross-linking, we do not know whether these mAbs also trigger a conformational change or impose an orientation of the LYVE-1 HABD that is favorable to HA binding, as has been proposed for antibodies, such as IRAWB14, that potentiate HA binding to CD44 (72, 73). However, this seems unlikely because all of the non-blocking LYVE-1 mAbs we tested enhanced HA binding, whereas only two from the large number of CD44 mAbs that have been identified to date display such properties (53). It is interesting to note that LYVE-1 can also form disulfide-linked homodimers (74),^3^ and it is possible that their cross-linking by the antibodies described here may also contribute to clustering through increased valency.

Whether cell intrinsic or extracellular factors may exert physiological LYVE-1 clustering and influence HA binding avidity *in vivo* is not yet clear. However, it is tempting to speculate that potential cross-linking molecules, such as the carbohydrate-binding galectins, might fulfill such a role, given reports that galectin 9 can interact with CD44 and influence HA binding (75, 76). Indeed, lymphatic endothelial cells themselves express several galectins (77),^4^ and evidence suggests that galectin 1 can also regulate cell migration in lymph (78).

In addition to factors that influence receptor clustering, we demonstrated for the first time that organization of the glycosaminoglycan within multimers or cross-linked supramolecular protein complexes can also facilitate stable binding to native LYVE-1. Specifically, we showed that prior cross-linking of HA as bHA·streptavidin multimeric complexes or preassociation of HA with the inflammation-induced hyaladherin TSG-6 (40, 41) elicited extensive binding to LYVE-1 on the surface of primary HDLEC. Importantly, unlike free HMW HA polymers, these higher order complexes appeared to bind without the requirement for prior receptor cross-linking. Thus, despite the innate multivalency of HA polymers, their additional cross-linking and/or conformational alteration appears to be obligatory for stable LYVE-1 engagement, most likely by increasing the capacity of the complexes themselves to induce LYVE-1 clustering. Consistent with this notion, the potentiation of HA binding that we observed using TSG-6 was much greater with the full-length protein (rhTSG-6) than its isolated HA-binding domain (Link_TSG6). Of note, previous studies have shown that the interaction with HA induces oligomerization of rhTSG-6 but not of Link_TSG6, allowing for protein-protein binding (possibly mediated by the CUB_C domain) that can cross-link HA chains (40). Hence, the differences in oligomerization properties between intact TSG-6 and Link_TSG6 may explain their differences in enhancing LYVE-1 HA interactions. Furthermore, bound bHA·streptavidin and bHA·rhTSG-6 complexes in both cases adhered to the surface of HDLEC in discrete puncta rather than in an even distribution on the cell surface. In further support of HA complex-induced clustering, we have found that Latex beads coated to high density with end-immobilized 300-kDa HA bind avidly to LYVE-1 in HDLEC and that adherent beads have discrete ringlike arrangements of the receptor around the contact zone (not shown). Moreover, as reported in our recent studies with HA-encapsulated Group A streptococci, adhesion to the surface of native lymphatic endothelial cells is associated with the formation of discrete LYVE-1 clusters beneath individual microbes (37). It should also be noted that in all of these cases, the avidity of HA binding and its relationship to LYVE-1 clustering will probably be influenced by HA size, a feature already documented for CD44 interactions but one that we did not explore in this current work.

The necessity of enforcing LYVE-1 clustering in order to achieve free HMW HA binding suggests that the native receptor may be constrained or physically restricted from dynamic self-association in lymphatic endothelium. At the very least, the effects of sialylation would be expected to prevent the formation of close contacts between neighboring LYVE-1 molecules through mutual charge repulsion. However, it is also possible that LYVE-1 is physically confined in the plasma membrane by anchorage to the underlying actin cytoskeleton, a known property of CD44 in some cell types and one that appears to regulate ligand binding (79–81). Although the cytoplasmic tail of LYVE-1, unlike that of CD44, lacks a consensus binding motif for ERM proteins or ankyrin (12, 13, 82, 83), we have observed that the majority of the receptor in HDLEC associates with the insoluble cytoskeleton fraction after appropriate lysis in non-ionic detergent. Indeed, preliminary evidence suggests that pharmacological disruption of the actin cytoskeleton in HDLEC monolayers may enhance HMW HA binding.^5^ Hence, the competence of native LYVE-1 to bind particular configurations of HA may be limited by the extent of its lateral mobility within the plasma membrane.

The finding that complex formation with TSG-6 augments HA binding to LYVE-1 in lymphatic endothelium has important implications for the physiological function of the receptor. Among its many important roles in tissue homeostasis, TSG-6 is frequently induced in inflamed tissues, including the synovium of osteoarthritic joints (42, 84), and its capacity to form complexes with HA is known to enhance binding to CD44 on the surface of transfected AKR1 T lymphoma cells (39). Furthermore, TSG-6 plays a catalytic role in formation of SHAP·HA (serum-derived hyaluronan-associated protein), a covalent adduct of HA and the heavy chain of inter-α-proteinase inhibitor (IαI) that is a constituent of HA aggregates deposited in inflamed tissues (85–89). Moreover, supramolecular complexes of HA with TSG-6, IαI heavy chain, and other binding partners, such as versican and pentraxins, that stabilize matrix HA in the form of cable structures have been shown to bind avidly to CD44 on monocytes that lack the competence to bind free glycosaminoglycan (56, 90–94). These parallels with CD44 raise the possibility that LYVE-1 could play a role in sensing HA complexes generated during inflammation *in vivo*, allowing the lymphatics to decode the status of interstitial matrix in injury or infection. Indeed, given that the lymphatics are a major route for clearance of HA from tissues for terminal degradation in lymph nodes (8, 10, 95), it is possible that LYVE-1 mediates not only binding but also uptake of such complexes by lymphatic endothelium. It should be noted that whereas LYVE-1 in its constitutively active state can internalize free high molecular weight HA in transfected 293T cells (13), no such activity has been demonstrated for LYVE-1 in its native “masked” state in LECs (15). Clearly, further experiments will be required to assess the true potential for endocytosis and subsequent degradation of HA supramolecular complexes by primary lymphatic endothelium *in vivo*.

Finally, the studies in this paper provide the first evidence that HA organized on the surface of macrophages, like that in the surface capsule of Group A streptococci, can also engage with LYVE-1 in native untransfected HDLEC and support both leukocyte adhesion and transendothelial migration *in vitro*. Such binding characteristics are in line with the role that we originally proposed for the LYVE-1·HA axis in mediating cell trafficking in the lymphatic network (11, 20), based on the discrete location of LYVE-1 in the characteristic button-like junctions of initial lymphatic capillaries that constitute sites for leukocyte transit *in vivo* (26, 38). In addition to the studies on macrophage HA in the present paper, it should be noted that a number of previous publications have documented the expression of HA synthases and hyaluronidases by human and murine macrophages and reported the synthesis not only of HA but also of versican and TSG-6 by primary macrophages and macrophage lines *in vitro* and *in vivo* (57, 61). Indeed, evidence has also been presented that dendritic cells can assemble an HA surface glycocalyx that mediates interactions with T cells important for antigen activation (58). In additional studies,^6^ we have confirmed the presence of HA on the surface of human MDM by fluorescent imaging with biotinylated hyaluronan-binding protein, particularly after LPS and INFγ activation, and have also detected HAS2 mRNA in this same cell population by RT-PCR. Clearly, however, further experiments will be required to determine precisely how this HA “coat” is retained on the macrophage surface, how it is modulated during macrophage differentiation and inflammation, and whether complex formation with binding partners, such as TSG-6 or IαI heavy chain, enhance its ability to bind native LYVE-1 in lymphatic endothelium *in vivo*. Given our finding that MDM adhesion and transmigration were greatly potentiated by mAb-induced LYVE-1 clustering *in vitro*, it seems reasonable to speculate that appropriate complexing of HA with its binding partners in the macrophage plasma membrane might lead to similar enhancement in an authentic inflammatory environment. Ongoing studies in our laboratory investigating the effects of LYVE-1 gene deletion and functional blockade on HA-mediated leukocyte migration and HA homeostasis in the lymphatics should ultimately shed light on these important issues.

In conclusion, our studies provide a new and clearer understanding of the critical factors required for stable interaction of LYVE-1 with HA in native lymphatic endothelium. These reveal LYVE-1 as a low affinity receptor whose dependence on multiple weak interactions for HA binding demands higher order multimerization of its glycosaminoglycan ligand and subsequent receptor clustering. Such properties may render this key receptor with the capacity to discriminate between different HA configurations and predict that LYVE-1 may play a role in selectively binding HA complexes associated with inflammation and in facilitating HA-mediated leukocyte trafficking *in vivo*.

## Author Contributions

D. G. J. conceived and coordinated the study, interpreted the data, and wrote the manuscript. W. L., S. Banerji, and S. Bhattacharjee designed, performed, and critically analyzed the experiments and interpreted the results. A. J. D. provided reagents for the experiments in Figs. 7 and 8 and helped to interpret the results. All authors reviewed the data and approved the final version of the manuscript.

## Footnotes

3 S. Banerji and D. G. Jackson, unpublished results.

4 L. A. Johnson and D. G. Jackson, unpublished results.

5 T. Stanly, C. Eggeling, and D. G. Jackson, unpublished results.

6 S. Bhattacharjee and D. G. Jackson, unpublished results.

## Footnotes

2 The abbreviations used are:

HA: hyaluronan
bHA: biotinylated hyaluronan
HMW: high molecular weight
HDLEC: human dermal lymphatic endothelial cell(s)
IαI: inter-α-trypsin inhibitor
MDM: monocyte-derived macrophage(s)
EDAC: *N-*(3-dimethylaminopropyl)-*N*′-ethylcarbodiimide hydrochloride
rhTSG-6: full-length recombinant human TSG-6
892: 891, and 893, commercial non-HA-blocking hLYVE-1 mAb 20892 and commercial HA-blocking mAbs 20891 and 20893, respectively
hLYVE-1: human LYVE-1
SA647: streptavidin Alexa 647
CMFDA: 5-chloromethylfluorescein diacetate
Ab: antibody.
